# The impact of heat waves on mortality in 9 European cities: results from the EuroHEAT project

**DOI:** 10.1186/1476-069X-9-37

**Published:** 2010-07-16

**Authors:** Daniela D'Ippoliti, Paola Michelozzi, Claudia Marino, Francesca de'Donato, Bettina Menne, Klea Katsouyanni, Ursula Kirchmayer, Antonis Analitis, Mercedes Medina-Ramón, Anna Paldy, Richard Atkinson, Sari Kovats, Luigi Bisanti, Alexandra Schneider, Agnès Lefranc, Carmen Iñiguez, Carlo A Perucci

**Affiliations:** 1 Department of Epidemiology, Regional Health Authority, Rome, Italy; 2 WHO Regional Office for Europe, Rome, Italy; 3 Department of Hygiene and Epidemiology, Medical School, University of Athens, Greece; 4 Centre for Research in Environmental Epidemiology, Barcelona, Catalonia, Spain; 5 National Institute of Environmental Health, Budapest, Hungary; 6 Division of Community Health Sciences, St. George's University, London, UK; 7 Public and Environmental Health Research Unit, London School of Hygiene and Tropical Medicine, London, UK; 8 Department of Epidemiology, Health Authority Milan, Milan, Italy; 9 Helmholtz Zentrum München, German Research Center for Environmental Health, Institute of Epidemiology, Neuherberg, Germany; 10 Department of Environmental Health, French Institute for Public Health Surveillance, Saint Maurice, Cedex France; 11 Epidemiology and Statistics Unit, Valencian School of Health Studies, Valencia, Spain

## Abstract

**Background:**

The present study aimed at developing a standardized heat wave definition to estimate and compare the impact on mortality by gender, age and death causes in Europe during summers 1990-2004 and 2003, separately, accounting for heat wave duration and intensity.

**Methods:**

Heat waves were defined considering both maximum apparent temperature and minimum temperature and classified by intensity, duration and timing during summer. The effect was estimated as percent increase in daily mortality during heat wave days compared to non heat wave days in people over 65 years. City specific and pooled estimates by gender, age and cause of death were calculated.

**Results:**

The effect of heat waves showed great geographical heterogeneity among cities. Considering all years, except 2003, the increase in mortality during heat wave days ranged from + 7.6% in Munich to + 33.6% in Milan. The increase was up to 3-times greater during episodes of long duration and high intensity. Pooled results showed a greater impact in Mediterranean (+ 21.8% for total mortality) than in North Continental (+ 12.4%) cities. The highest effect was observed for respiratory diseases and among women aged 75-84 years. In 2003 the highest impact was observed in cities where heat wave episode was characterized by unusual meteorological conditions.

**Conclusions:**

Climate change scenarios indicate that extreme events are expected to increase in the future even in regions where heat waves are not frequent. Considering our results prevention programs should specifically target the elderly, women and those suffering from chronic respiratory disorders, thus reducing the impact on mortality.

## Introduction

Several studies in the United States and Europe have shown marked short-term increases in the number of deaths during heat waves, however the role of these extreme events on mortality needs to be better clarified [1]. Climate change projections for Europe show that over the next century, heat waves will become more frequent, intense and will last longer, not only in Mediterranean regions, but also in Northern areas currently not characterized by heat wave events [2]. These changes could contribute to the burden of disease and premature deaths, particularly in vulnerable populations with limited adaptation resources [3].

In meteorological terms, a heat wave is defined as a prolonged period of unusually hot weather. To date, a standard definition of heat wave has not been agreed upon and different definitions have been used to evaluate its impact on health [4-7].

During summer 2003, Europe experienced one of the worst heat wave events in recent history [8], with an estimated excess mortality varying between 25.000 and 70.000 deaths in Western Europe [9,10]. The comparison of the impact of the 2003 heat wave between countries is hampered by the substantial differences in the methodologies employed to define heat wave events and to estimate the excess mortality [4,6,11-13].

The present paper presents the main results of the EuroHEAT project (Improving Public Health Responses to extreme weather/heat-waves), which aimed to develop a standardized definition of a heat wave event and to compare the impact of heat waves in European cities. The study took advantage of the database and the findings of a previous European collaborative study (Assessment and Prevention of acute Health Effects of Weather conditions in Europe - PHEWE), which estimated the impact of high temperatures on mortality using a time series approach [14]. The methodology developed within EuroHEAT meets the need to investigate the specific contribution of single heat wave events on mortality and allows for a valid comparison between cities.

Given the evidence that heat waves do not affect the entire population but susceptibility is greater among the elderly [3,4,7], EuroHEAT was focused on people aged 65 years and over.

The main objectives of this paper are:

• to provide a unique definition of "heat wave" to be applied across cities in the subsequent analyses;

• to estimate the health impacts of heat wave episodes on the elderly population, by gender, specific causes and age groups;

• to evaluate the role of specific heat wave characteristics, namely duration, intensity and timing within the season on mortality;

• to compare the impact of the 2003 heat wave across nine European cities using the same methodological approach.

## Methods

### Study population and setting

The EuroHEAT project involves nine European cities (Athens, Barcelona, Budapest, London, Milan, Munich, Paris, Rome, Valencia), with a total population of around 25 million citizens, which represent a variety of climatic, socio-economic, and air pollution characteristics. Available data for daily mortality, meteorological and air pollution were provided for each city between 1990 and 2004. Only summer months (June-August) were included in the study. Summer 2003 was analyzed separately to assess the impact of this exceptional heat wave episode that affected most of the European cities, and results were compared with heat waves from other years included in the study period.

### Data base

Mortality data were daily counts of primary deaths for all natural causes (ICD-9: 1-799; ICD-10: group A-R), cardiovascular (ICD-9: 390-459; ICD-10: group I), cerebrovascular (ICD-9: 430-438; ICD-10: group I 600-699), and respiratory causes (ICD-9: 460-519; ICD-10: group J), by gender and age groups (65-74, 75-84, 85 +), except from Paris where only total mortality was available in 2003.

Meteorological data including air temperature, dew point temperature, sea level pressure, total cloud cover, wind speed and wind direction were collected for the entire study period at the city airports, every 3 hours.

To investigate potential confounding by air pollution, data were also collected for the following variables: SO_2 _(24-hour mean), TSP or Black Smoke (24-hour mean), PM10 (24-hour mean), PM2.5 if available (24-hour mean), NO_2 _(maximum 1 hour, 24-hour mean), O_3 _(maximum 1 hour, maximum 8-hour moving average), and CO (maximum 8-hour moving average).

### Exposure definition

Exposure to heat waves considered both extreme day time values in terms of maximum apparent temperature (Tappmax) and high night time temperatures through minimum temperature (Tmin).

Tappmax is a discomfort index based on air and dew point temperature, calculated using the following formula [15,16]:

$$T_{app}=-2.653+0.994\left(T_{air}\right)+0.0153{\left(T_{dewpt}\right)}^{2}$$

Heat waves were thus defined as

1) periods of at least two days with Tappmax exceeding the 90^th ^percentile of the monthly distribution

or

2) periods of at least two days in which Tmin exceeds the 90^th ^percentile and Tappmax exceeds the median monthly value.

Daily counts of total and cause specific mortality (cardiovascular, cerebrovascular and respiratory) in the heat wave periods were considered as outcome variable, stratifying by gender and age groups (65-74 yrs, 75-84 yrs, 85 + yrs).

### City specific analysis

As the first stage, a city specific analysis was conducted using Generalized Estimating Equations (GEE models)[17] to analyze longitudinal data. A Poisson distribution was assumed for the outcome variable (mortality) and days were characterized as "heat wave" or "non heat wave" days as the exposure variable to estimate the effect on mortality.

Observations from different years were assumed to be independent, while observations within the same summer were correlated. A similar approach has already been suggested in other studies [18-21]. Since the number of clusters (summers) was small, and equal to the number of years in the study period, we used the model-based estimator for the coefficients' standard errors, as recommended in the presence of few large clusters [22]. A first order autoregressive structure within each year was chosen, based on an exploratory analysis similar to the one described by Chiogna & Gaetan [23,24].

A common model was specified for each city taking into account the following as potential confounders: holidays, day of the week and calendar month, linear terms for barometric pressure (lag 0-3) and wind speed, linear and quadratic terms for time trend and the maximum 1-hour daily value of NO_2 _(μg/m^3^) at (lag 0-1). NO_2 _was chosen to adjust for air pollution as it has shown to be a good indicator of traffic in a large European collaborative project [25] which assessed the impact of air pollution on mortality, using meteorological variables as potential effect modifiers.

The effect was estimated as the percent increase in daily mortality during heat wave compared to non heat wave days. The effect of each heat episode was also investigated with respect to specific heat wave characteristics such as *duration, intensity *and *timing *within the season.

Duration was categorized using the city specific median values of heat wave duration (number of consecutive heat wave days) as the cut-point:

- *short heat wave *if duration was shorter than the median,

- *long heat wave *if duration was equal to or longer than the median.

Intensity was also categorized into two levels according to the extreme values of Tappmax reached during the heat wave:

- *low intensity heat wave *if Tappmax was below the monthly 95^th ^percentile,

- *high intensity heat wave *if Tappmax was equal to or above the monthly 95^th ^percentile.

Timing within the season was defined according to the time interval between different heat waves:

- *the first heat wave *of each summer was considered separately,

- *heat waves *that occurred between 1 and 3 days after the previous one,

- *heat waves *that occurred three or more days after the previous one.

### Pooled analysis

To summarize city-specific results, cities were grouped into two regions, according to geographical and climatological criteria in order to control for heterogeneity, as defined in the PHEWE study [18]: "Mediterranean" (Athens, Barcelona, Milan, Rome, and Valencia) and "North-Continental" (Budapest, London, Munich and Paris) City-specific estimates were combined through a random effect meta-analysis using the method described by DerSimonian and Laird [26]. To estimate the impact in each region, a GEE model was used, similar to the city-specific model, but adding a city indicator variable and interaction terms of the exposure variable with the confounders.

## Results

### Heat wave definition

The appropriateness of the heat wave definition was tested on the 2003 time series data, as this was the year when heat waves were observed in almost all cities. For all cities, the definition was able to capture consecutive days with high temperatures, excluding only isolated peaks.

Table 1 presents datasets and the city characteristics. The number of natural deaths per day in the age group 65 + ranged from 12 in Valencia to 117 in London. Cardiovascular causes accounted for about 40-50% of the total, except in Paris (34.1%), Budapest (60.9%) and Athens (51.6%). Cerebrovascular mortality was about 10% of total mortality, with the lowest proportion observed in Munich and Valencia (8.3%) and the highest in Athens (17.7%). Great heterogeneity was observed in respiratory deaths, generally accounting for less than 10% of the total mortality, with the highest proportion in London (16.2%) and the lowest in Budapest and Munich (4.3%). Mortality data for specific causes of death were not available for Paris in 2003.

**Table 1:** City characteristics, mean daily number of deaths by cause (count and % of total), Maximum Apparent Temperature, Minimum Temperature and number of annual heat wave days during summer (June-August) in the study period.

City	Study period	Population size (65+ years)	All natural deaths	Respiratory deaths	Cardiovascular deaths	Cerebrovascular deaths	Minimum temperature (°C) 90th percentile		Maximum apparent temperature (°C) 90th percentile		Heat wave days	
			
			n	n (%)	n (%)	n (%)	All years°	2003	all years°	2003	all years°	2003
**Athens**	1997 - 2004	505935	62	5 (8.1)	32 (51.6)	11 (17.7)	26.4	27.3	37.8	37.6	4 - 21	11
**Barcelona**	1991 - 2004	331665	30	3 (10.0)	12 (40.0)	4 (13.3)	23.2	27.0	33.1	36.8	5 - 10	43
**Budapest**	1992 - 2003	300940	46	2 (4.3)	28 (60.9)	7 (15.2)	21.6	22.6	31.6	32.8	3 - 18	16
**London**	1990 - 2004	937000	117	19 (16.2)	49 (41.9)	11 (9.4)	16.8	18.1	27.1	30.8	3 - 27	14
**Milan**	1990 - 2003	281024	21	2 (9.5)	9 (42.9)	3 (14.3)	22.2	25.0	36.2	42.2	3 - 14	45
**Munich**	1992 - 2004	193246	24	1 (4.2)	12 (50.0)	2 (8.3)	15.9	18.1	28.3	31.9	3 - 15	30
**Paris**	1990 - 2003	809061	82	6 (7.3*)	28* (34.1)	7 (8.5*)	19.1	22.4	30.9	35.0	3 - 15	18
**Rome**	1992 - 2004	306570	42	3 (7.1)	18 (42.9)	5 (11.9)	21.6	23.8	35.2	36.2	3 - 15	30
**Valencia**	1994 - 2003	128668	12	1 (8.3)	4 (33.3)	1 (8.3)	24.2	25.6	39.9	41.7	4 - 13	32

*cause of deaths not available in 2003

° except 2003

Temperature indicators showed a large variability of the meteorological conditions. Considering all years in study, the range for minimum temperature was from 15.9 °C in Munich to 26.4 °C in Athens, and for maximum apparent temperature from 27.1 °C in London to 39.9 °C in Valencia. In every city the number of heat waves days was heterogeneous among years. In 2003, an increase in both minimum and maximum apparent temperature was observed in all cities, except in Athens. The number of heat wave days was generally higher in 2003 in all cities except in Athens, Budapest and London.

### Health impacts of heat waves

Table 2 showed the heat wave effect in the study period (1990-2002 and 2004), estimated as the percentage increase in mortality *per day *with respect to non heat waves days for all natural deaths, respiratory, cardiovascular and cerebrovascular mortality. During heat waves days a significant increase in total daily mortality was observed in all cities, with the greatest increase in Milan (+ 33.6%, 95%CI + 28.5%-39.0%) and the lowest in Munich (+ 7.6, 90% C.I. + 3.8-11.5). A significant increase in daily mortality was observed for all the causes of death considered, except for respiratory mortality in Munich. In most cities the highest increment was observed for respiratory causes.

**Table 2:** City specific estimates of the effect of heat waves on daily mortality (% increase and 90% CI) by cause of death among people aged 65+ years.

	All natural deaths		Respiratory deaths		Cardiovascular deaths		Cerebrovascular deaths	
				
City	% increase	(90% CI)	% increase	(90% CI)	% increase	(90% CI)	% increase	(90% CI)
**Athens**	21.6	(18.5 - 24.8)	34.5	(24.6 - 45.2)	28.4	(24.0 - 33.0)	33.0	(25.9 - 40.4)
**Barcelona**	15.6	(11.0 - 20.4)	41.3	(26.4 - 57.9)	21.4	(14.0 - 29.4)	25.1	(12.4 - 39.3)
**Budapest**	21.1	(17.3 - 24.9)	20.6	(2.6 - 41.7)	24.1	(19.3 - 29.1)	24.6	(15.6 - 34.4)
**London**	10.4	(8.6 - 12.2)	18.0	(13.4 - 22.8)	9.3	(6.6 - 12.1)	10.6	(5.2 - 16.3)
**Milan**	33.6	(28.5 - 39.0)	92.5	(72.3 - 115.1)	39.2	(31.2 - 47.6)	49.8	(35.6 - 65.6)
**Munich**	7.6	(3.8 - 11.5)	3.9	(-0.8 - 30.8)	8.2	(2.8 - 13.9)	14.7	(2.4 - 28.6)
**Paris**	11.4	(10.0 - 12.9)	27.7	(19.4 - 36.6)	12.3	(8.5 - 16.2)	19.7	(12.1 - 27.8)
**Rome**	26.8	(23.4 - 30.4)	66.9	(51.9 - 83.3)	37.8	(32.5 - 43.3)	48.0	(38.0 - 58.8)
**Valencia**	8.5	(1.2 - 16.3)	32.4	(9.1 - 60.7)	20.1	(7.9 - 33.6)	1.4	(-17.4 - 24.4)

Table 3 summarize the pooled results for North continental and Mediterranean cities, by gender, age groups, and cause of death (all causes, respiratory, cardiovascular, and cerebrovascolar causes). The effect was higher in Mediterranean cities than in North-Continental cities for all groups of causes considered. In both two groups of cities the highest increase in daily mortality was observed for respiratory causes.

**Table 3:** Pooled estimates of the effect of heat waves on daily mortality (% increase and 90% CI) by gender, cause of death and age groups.

	North-Continental cities				Mediterranen cities			
		
	Males		Females		Males		Females	
				
	% increase	(90% CI)	% increase	(90% CI)	% increase	(90% CI)	% increase	(90% CI)
**All causes**								
**65-74 age group**	8.2	(5.2 - 11.3)	9.7	(6.2 - 13.4)	14.5	(10.4 -18.5)	23.0	(17.8 -28.4)
**75-84 age group**	12.4	(9.7 - 15.3)	14.9	(12.3 -17.7)	18.1	(14.2 -21.9)	33.0	(28.9 -37.3)
**85+ age group**	10.7	(7.3 - 14.3)	18.4	(16.0 -21.0)	32.3	(27.1 -37.9)	35.9	(31.9 -40.1)
								
**Respiratory**								
**65-74 age group**	16.8	(6.3 - 28.1)	12.6	(-0.1 -27.0)	32.4	(14.3 -53.3)	45.8	(22.3 -73.7)
**75-84 age group**	19.0	(11.0 -27.6)	21.4	(12.9 -30.6)	44.8	(30.9 -60.0)	61.3	(44.3 -80.4)
**85+ age group**	12.1	(3.5 - 21.4)	30.0	(22.8 -37.6)	58.9	(42.9 -76.5)	58.1	(44.3 -73.3)
								
**Cardiovascular**								
**65-74 age group**	6.8	(2.2 -11.7)	12.3	(6.3 -18.6)	14.7	(7.8 -22.0)	38.0	(28.7 -48.0)
**75-84 age group**	9.7	(5.5 -14.0)	15.7	(11.7 -19.8)	18.4	(12.4 -24.7)	43.3	(37.0 -50.1)
**85+ age group**	10.6	(5.2 -16.3)	17.5	(14.0 -21.2)	34.7	(27.1 -42.8)	38.5	(33.4 -43.9)
								
**Cerebrovascular**								
**65-74 age group**	6.3	(-4.6 -18.5)	12.2	(-0.3 -26.4)	31.9	(16.3 -49.5)	37.9	(21.4 -56.7)
**75-84 age group**	11.6	(2.8 -21.3)	20.4	(12.7 -28.7)	20.3	(8.9 -32.8)	44.1	(33.5 -55.4)
**85+ age group**	17.8	(6.5 -30.3)	19.4	(12.5 -26.5)	41.1	(27.1 -56.7)	39.5	(30.5 -49.2)

The effect of heat waves increased with age and a statistically significant higher impact among females than males was observed in Mediterranean cities in the 75-84 age group.

### Role of specific heat wave characteristics

**Figure 1:**
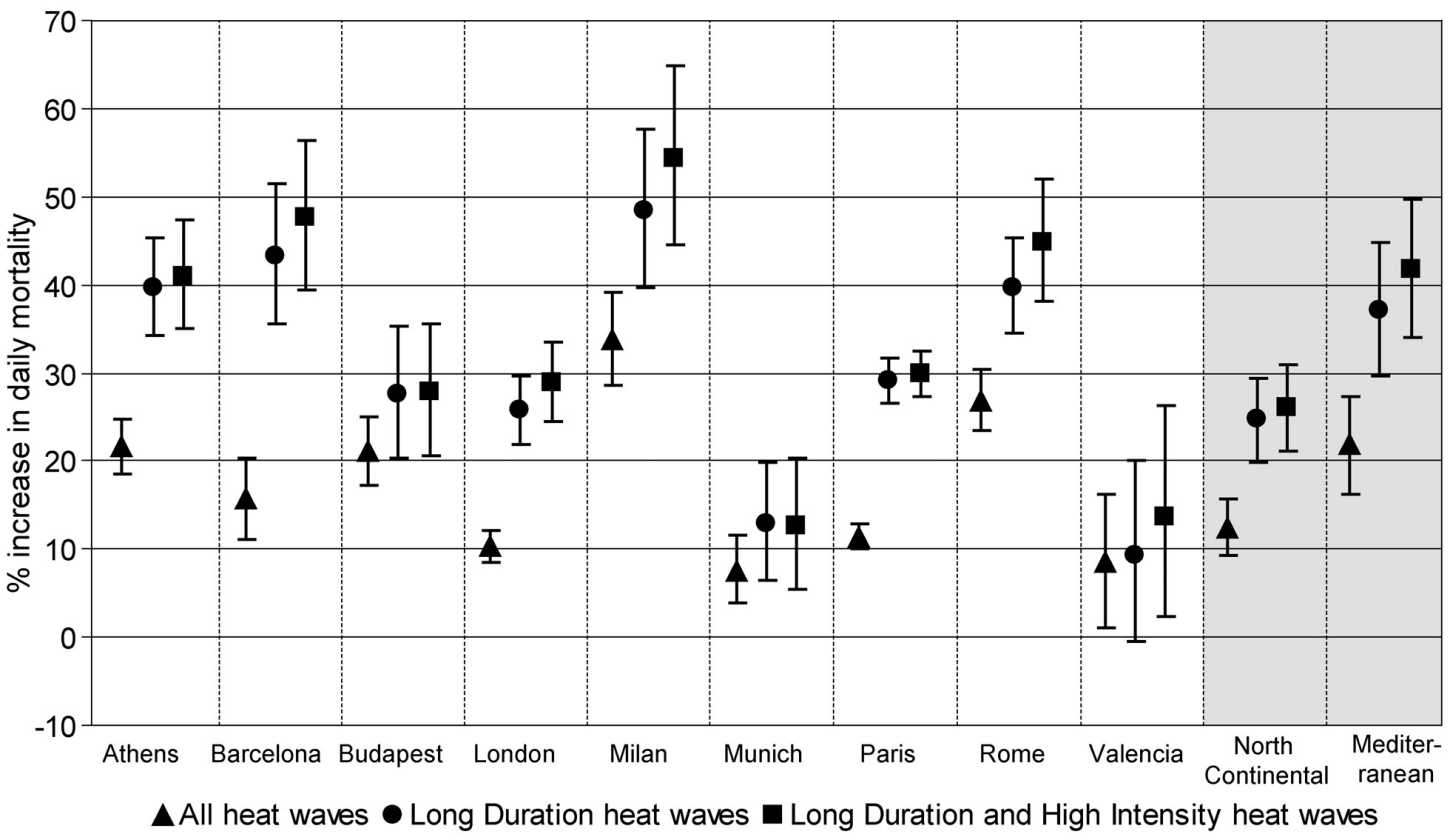
**City-specific and pooled estimates of the effect of heat-waves on daily mortality (% increase and 90% CI) by duration and intensity**.

The effect of specific characteristics of heat waves in terms of duration and a combination of duration with intensity was investigated and results are shown in Figure 1. A higher effect of heat waves of longer duration and high intensity is shown in most cities. Results give evidence for duration to play a bigger role than intensity. In the pooled analysis, a two-fold impact of longer and high intense heat waves is observed in both European regions.

The analysis of the role of timing of heat waves did not produce homogeneous results across cities. The first heat wave of the summer had a greater impact only in some cities. In case of subsequent heat waves, those occurring after less than 3 days after the end of the preceding one had a weaker effect (data not shown).

### The impact of the 2003 heat wave

**Figure 2:**
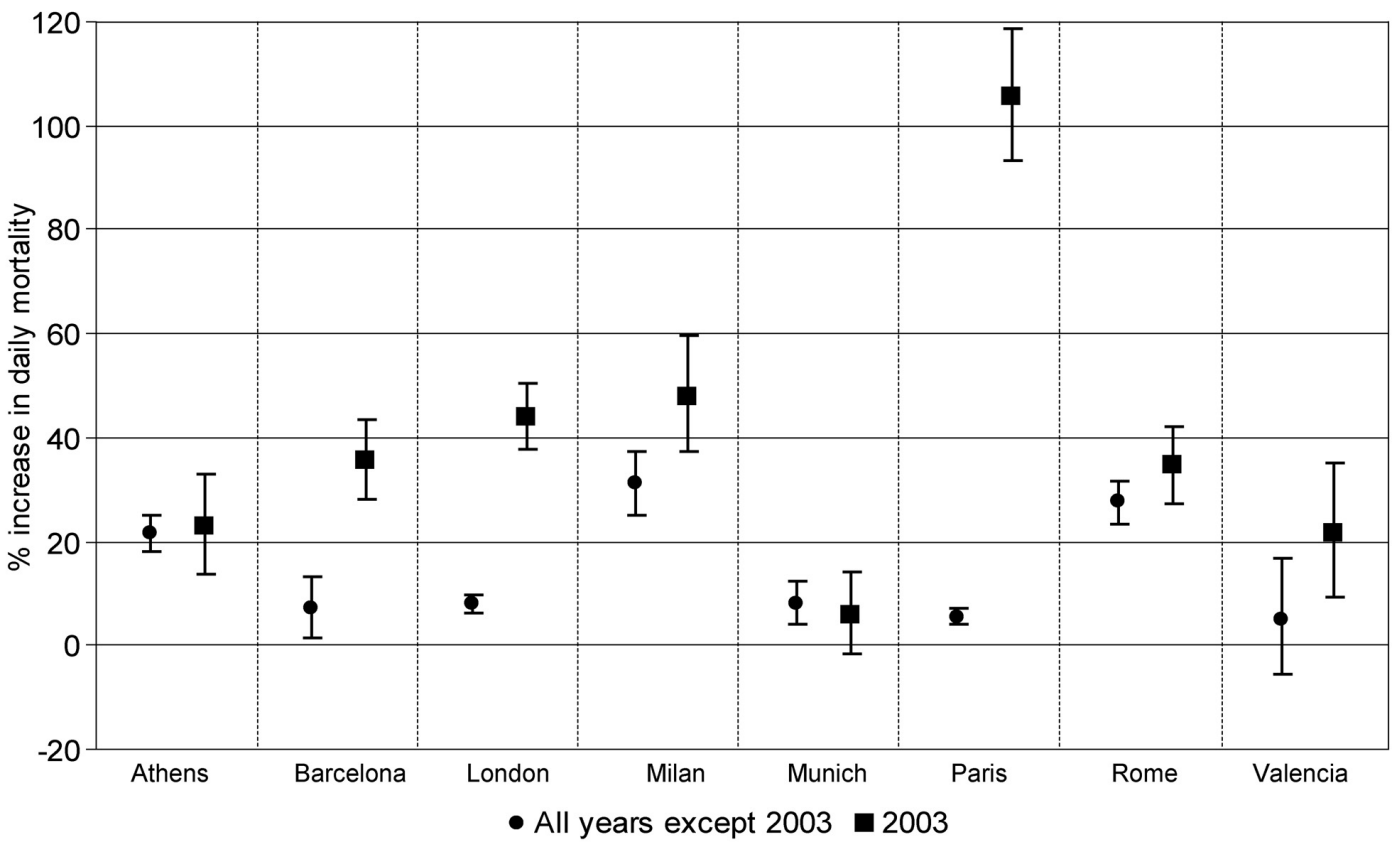
**City-specific estimates of the effect of heat-waves on daily mortality (% increase and 90% CI) during summer 2003 and in other years**.

Figure 2, compared the effect of heat waves in the entire study period and during summer 2003. In all cities, expect in Athens and Budapest, heat waves in 2003 were more extreme and showed an higher impact on daily mortality than the effect observed in the other years. The biggest difference was observed in Barcelona (+ 7.1%, 90% C.I.1.4-13.0 and + 35.7, 90% C.I. 28.3-43.5), London (+ 7.9%, 90% C.I. 6.1-9.8 and + 43.8%, 90% C.I. + 37.6%,50.3%) and Paris (+ 5.5%, 90% C.I: 3.9%-7.2%, and + 105.5%, 90% C.I. + 93.2%- + 118.7%).

## Discussion

This study is the first in Europe to compare the impact of heat waves on mortality in different cities using a common heat wave definition and a standardized methodological approach. Heat wave episodes were defined taking into account both temperature and humidity levels during the day as well as high night-time temperatures, thus accounting for the physiological impact of heat on health [27]. Moreover, the impact on mortality was also evaluated considering specific heat wave characteristics; results showed that the intensity, duration and timing of heat waves influenced the risk of mortality during extreme events. Heat waves of long duration had the greatest impact on mortality, and resulted in 1.5 to 3 times higher daily mortality than for other heat waves.

These results together with findings from the previous study (PHEWE project) conducted in the same cities [18], using a time series approach, provide a complete overview of the effect of high temperature in European cities.

Results showed great heterogeneity in the effect of heat waves on daily mortality. In the study period, the strongest impact was observed in the Mediterranean cities where heat waves are more frequent and characterised by higher temperatures, confirming results from the PHEWE project time series analysis [18].

In contrast, in 2003 the greatest impact on mortality was observed in cities where heat wave episodes are rare events or were characterized by temperatures largely outside the usual meteorological conditions. In several cities, the 2003 episodes were characterized by more extreme temperatures, with both maximum and minimum temperature several degrees above the average values. However, differences in exposure alone do not seem to explain the greater impact observed in North-Continental than in Mediterranean cities. A poor ability to adapt to high temperatures among residents in North-Continental cities may be the explanation for the highest impact on these populations, as observed in France in 2003 [28].

We found that the impact on daily mortality increases with age. Moreover, gender was among the factors that increases individual vulnerability to heat waves; we observed a higher susceptibility of females even after stratifying by age groups. Other authors observed similar results [9,10,12] and this finding may be attributable to social conditions of elderly women living alone and to physiological differences, such as a reduced sweating capacity that affects the ability to respond to heat stress [9]. It may also be due to residual confounding of age, as within the older age groups there will be a greater number of women than men.

In most cities, a greater effect of heat waves on respiratory than on cardiovascular mortality was observed. This is in agreement with other studies [7,29] and probably reflects the fact that health status of people suffering from chronic respiratory diseases rapidly deteriorates during hot periods or that the capacity to cope with heat stress is generally diminished in particular population subgroups, such as the elderly [30,31]. However, the underlying mechanisms still remain unclear [32].

Several population characteristics like social isolation and income level have been identified as factors that affect the susceptibility of urban populations to heat, as have the adaptive measures and actions in place [33]. However, information on these characteristics were not available in the present study.

A limit of the analysis is that neither the lag effect, nor the role of harvesting could be investigated. A relevant question that still remains unclear is whether a reduction in mortality is observed shortly after a severe heat episode. Results from the PHEWE study [18] showed some evidence of a harvesting effect both in the Mediterranean and in the North-Continental cities, although the observed excess in mortality associated with high temperatures was only partially compensated for by mortality displacement. Previous studies on heat wave episodes have also reported a compensatory decrease in overall mortality during the weeks following a heat wave [9,34-36]; however, the study on the impact of the 2003 heat wave in 9 French cities [28] did not report any short-term mortality displacement. Differences in exposure intensity and the differential impact of mortality on susceptible subgroups may explain why short-term mortality displacement is reported in some studies [9,34-36] and not in others [28].

Considering the latest IPCC Assessment Report, climate change predictions for Europe show an increase in the frequency and the intensity of heat waves, especially in central, southern and eastern areas [3] and as consequence heat-related mortality will become a relevant threat even in cities usually not exposed to extreme hot temperatures. Considering our results prevention programs should specifically target the elderly, especially women, and those suffering from chronic respiratory disorders, in order to reduce in the future the burden of heat-related mortality.

## Authors' contributions

DD and PM contributed to defining the objectives of the analysis, designed and supervised the analysis the analysis and drafted the paper; CM contributed to data management and carried out the analysis; Fde'D and UK contributed to writing and revised the paper; BM, KK and AA contributed to defining the objectives of the analysis and to writing and revising the paper; MM, AP, RA, SK, LB, AS, AL, and CI contributed to collect and revised the paper; CAP participated to the discussion of methodology and results and revised the paper. All authors read and approved the final version.

## Competing interests

The authors declare that they have no competing interests.
